# Harnessing sub-comb dynamics in a graphene-sensitized microresonator for gas detection

**DOI:** 10.1007/s12200-024-00115-5

**Published:** 2024-05-01

**Authors:** Yupei Liang, Mingyu Liu, Fan Tang, Yanhong Guo, Hao Zhang, Shihan Liu, Yanping Yang, Guangming Zhao, Teng Tan, Baicheng Yao

**Affiliations:** 1 Key Laboratory of Optical Fiber Sensing and Communications (Ministry of Education), University of Electronic Science and Technology of China, Chengdu, 611731 China; 2 Institute of Semiconductors, Chinese Academy of Sciences, Beijing, 100083 China; 3 Engineering Center of Integrated Optoelectronic & Radio Meta-Chips, University of Electronic Science and Technology, Chengdu, 611731 China

**Keywords:** Microresonator, Optical frequency comb, Graphene, Gas sensing

## Abstract

Since their inception, frequency combs generated in microresonators, known as microcombs, have sparked significant scientific interests. Among the various applications leveraging microcombs, soliton microcombs are often preferred due to their inherent mode-locking capability. However, this choice introduces additional system complexity because an initialization process is required. Meanwhile, despite the theoretical understanding of the dynamics of other comb states, their practical potential, particularly in applications like sensing where simplicity is valued, remains largely untapped. Here, we demonstrate controllable generation of sub-combs that bypasses the need for accessing bistable regime. And in a graphene-sensitized microresonator, the sub-comb heterodynes produce stable, accurate microwave signals for high-precision gas detection. By exploring the formation dynamics of sub-combs, we achieved 2 MHz harmonic comb-to-comb beat notes with a signal-to-noise ratio (SNR) greater than 50 dB and phase noise as low as − 82 dBc/Hz at 1 MHz offset. The graphene sensitization on the intracavity probes results in exceptional frequency responsiveness to the adsorption of gas molecules on the graphene of microcavity surface, enabling detect limits down to the parts per billion (ppb) level. This synergy between graphene and sub-comb formation dynamics in a microcavity structure showcases the feasibility of utilizing microcombs in an incoherent state prior to soliton locking. It may mark a significant step toward the development of easy-to-operate, systemically simple, compact, and high-performance photonic sensors.

**Graphical Abstract:**

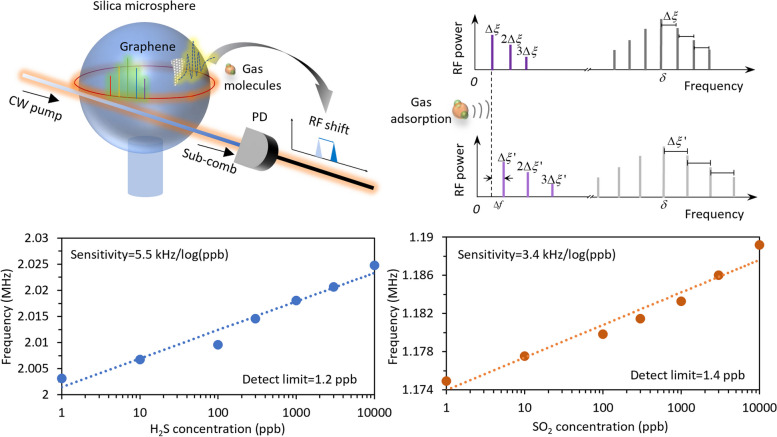

## Introduction

The advent of the optical frequency comb, featuring equidistant frequency components, has spurred revolutionary sciences in optoelectronics [1]. This innovation has led to a multitude of advancements, including the development of optical clocks for frequency-time calibration [2], precise spectrometers for biochemical detection [3], and coherent distributed detection systems for deep-sea oil and gas exploration [4]. Notably, microcombs, which leverage the Kerr effect in microresonators, open a way for integrating frequency combs and have emerged as a foundational element of modern photonic technology [5], demonstrating significant potential in areas such as frequency metrology [6], data transmission [7], photonic logic operation [8], optical computing [9], microwave photonics [10], and molecular sensing [11]. Particularly, soliton microcombs, valued for their stable and coherent output [12], attract most explorations. However, traditional solitons primarily exist within the thermally unstable red detuning interval. Addressing the thermal effects on soliton formation necessitates spontaneous and reliable methods, such as power-kicking [13] and auxiliary-assisted strategies [14], for soliton initiation and maintenance, which adds significant complexity to the system. This complexity diminishes the advantages of Kerr microcombs over mode-locking laser microcombs [15]. In contrast, certain incoherent regimes like sub-combs and chaotic states, which arise spontaneously from modulational instability (MI) in the blue detuning interval, offering thermal stability [16], are the nonlinear system's dominant attractors and can be excited more easily. Recent research has shown that these regimes can be harnessed for specific applications by utilizing their unique properties, such as employing chaotic combs for parallel, congestion-free ranging [17].

In this study, we explore the formation dynamics of sub-combs excited in a microresonator and leverage their time–frequency properties in gas sensing application. By precisely controlling the merging process of sub-combs, we successfully generate stable and accurate beating signals in the radio frequency domain through inter-comb heterodyne, which are easy to excite and decipher, in contrast to RF sensing probes generated by interleaved Raman solitons [11] or mode-splitting [18]. These signals not only exhibit a high signal-to-noise ratio (SNR) of up to 50 dB, but also maintain long-term stability, with a spectral shift of less than 500 Hz over a 10-min period. Moreover, we find that the sensitivity of the sub-comb-based beating signals can be activated by incorporating a layer of intracavity graphene. This modification overcomes the limitations of a pristine cavity [18], and thus imbues the signals with an exceptional frequency response to gas molecules. The underlying sensing mechanism is elucidated through theoretical analysis, complemented by numerical simulations based on the Lugiato-Lefever equation (LLE), and is confirmed experimentally. The self-beating signal generated from the sub-comb is observed at 2 MHz, with a 3 dB linewidth of 0.5 kHz. Utilizing the graphene-enhanced sub-combs, we demonstrate the precise detection of two types of gas molecules, H_2_S and SO_2_, with detect limits reaching as low as 1.2 and 1.4 ppb, respectively.

## Conceptual design and simulations

**Fig. 1:**
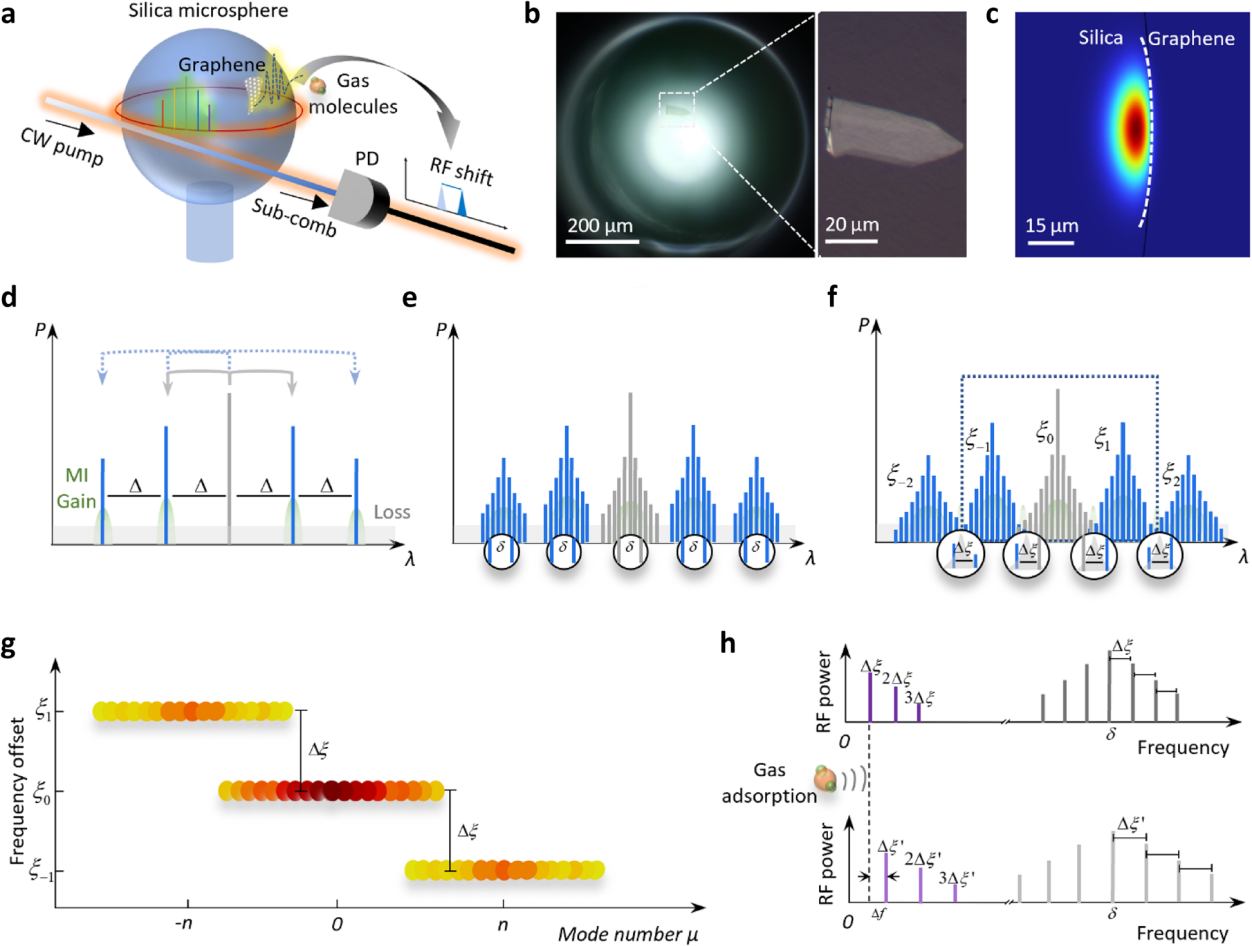
Sub-comb formation dynamics in a graphene-silica microresonator for gas sensing. **a** Schematic diagram shows sub-comb are generated in a graphene-sensitized microsphere, and self-beating of the sub-comb produces a radio frequency signal for gas detection. In this device, gas-graphene interaction leads to frequency shift of this beat note. **b** Microscopic pictures of the device. Diameter of the silica microsphere is ≈ 600 μm (left panel), and the graphene nanolayer is deposited on the microsphere, with a position 20° away from the resonant equator. Size of the graphene monolayer is 50 μm × 20 μm (right panel). **c** Simulated electric field distribution of the fundamental mode in the WGM microresonator. **d** Generation of primary combs with a frequency distance Δ due to degenerate FWM (gray arrows) and non-degenerate FWM (blue arrows). **e** Generation of sub-combs with equal frequency distance *δ* in each bunch. **f** Merging state of sub-combs. In the overlap region of adjacent bunches, more than one comb lines reside in one resonance, with interval Δ*ξ*. **g** Frequency offset of three bunches in the dashed box in **f**. Darker color represents higher comb line power. **h** Beating signals before and after gas absorption. The gas adsorption on graphene introduces a frequency shift Δ*f*

Figure 1a illuminates the conceptual design of our device. A piece of graphene nanolayer is attached to a silica microsphere. With external continuous wave (CW) pumping, the Kerr nonlinearity intracavity enables photonic modulation. During the red-tuning of pump frequency, sub-combs are generated, after the Turing state [19]. Specifically, there could be two comb lines co-excited in a single resonance. This enables self-beating in radio frequency, which can be directly measured in a photodetector (PD). Then, thanks to the graphene deposited on the microresonator, gas adsorption can influence the cavity’s group velocity dispersion (GVD) via effective index modification [20]. This changes the spacing of primary sidebands, leading to frequency shift of the beating line. The microscopic picture of our graphene-sensitized microsphere is shown in Fig. 1b. Diameter of the microsphere is 600 μm, corresponding to a free spectral range (FSR) about 100 GHz. The monolayer graphene nanosheet, with length of 50 μm and width of 20 μm, is mechanically exfoliated and dry-transferred onto the microsphere [21]. In this whispering gallery mode (WGM) microcavity, the graphene is deposited 20° away from the resonant equator, for avoiding thermal-damage due to the extremely high intracavity power. Specifically, the simulated fundamental electric field distribution of WGM in the cavity is presented in Fig. 1c, it illustrates that spatial area of the fundamental mode is ≈ 80 μm^2^. *Q* factor of this graphene functionalized microsphere is > 6.7 × 10^8^, suggesting an intracavity power density reaches 1.67 × 10^14^ W/cm^2^, when the input pumping power is 20 mW.

Figure 1d schematically depicts the generation of primary comb sidebands. When pump is injected into the cavity, parametric gain is produced via modulational instability (MI) [22]. When MI gain overcomes the intracavity loss in the cavity, the first pair of oscillating bands would grow from noise [23]. In this energy conservation process, frequency relation between pump and sidebands can be described as 2*ω*_p_ = *ω*_i_ + *ω*_s_, where *ω*_p_, *ω*_i_, and *ω*_s_ are angular frequencies of the pump, the idler, and the signal, respectively. In this case, the two oscillating frequencies are symmetrically distributed on each side of the pump, with a spectral distance Δ. This process is termed as parametric oscillation or degenerate four-wave mixing (FWM), shown as gray arrows. The distance Δ is usually greater than one FSR and can be mathematically expressed as [24]

1 $$\Delta = \sqrt {\frac{{4{\uppi }n_{{\text{g}}} (f_{{\text{p}}} - N\delta n_{{\text{g}}} /n_{0} )}}{{\beta_{2} c}} - \frac{{4\gamma P_{{\text{in}}} }}{{\beta_{2} }}} .$$

In Eq. (1), *n*_0_ is refractive index, *n*_g_ is group refractive index, *γ* is nonlinear coefficient, *P*_in_ is launch-in pump power, *c* is speed of light, *β*_2_ is GVD, *δ* is the FSR at pump mode, *N* is longitudinal mode number, which can be calculated by *N* = *f*_0_*n*_0_/*δn*_g_, where *f*_0_ is center frequency of the pumping resonance, and *f*_p_ is pump frequency.

Then, non-degenerate FWM process commences, which can be described by *ω*_1_ + *ω*_2_ = *ω*_3_ + *ω*_4._ The pump and first sidebands act as photon donors, deliver optical energy to other comb lines (blue arrows) with the same spacing Δ, forming primary combs. Since MI gain usually covers several FSRs, with intracavity pump power enhanced ulteriorly, secondary comb lines are generated around primary sidebands, forming several comb bunches (Fig. 1e). This state is termed as sub-comb, or bunched comb [25]. Although these bunches reside at spectral positions with different FSRs, their comb lines are natively spaced, with a spacing equal to *δ*. This can be explained as FWM imposing the same time-dependent refractive index modulation to all the modes [26]. When the intracavity pump power further increases, parametric growth of new frequency components would be spurred under the synergy of degenerate and non-degenerate FWM. As a result, the comb bunches spread out and finally overlap with each other (Fig. 1f). At this moment, more than one comb line might reside in the resonance among the superimposed region [27]. Frequency components in each bunch are deviate from an equidistant frequency grid with spacing *δ* to the origin at pump frequency. Therefore, the offset of *i*-th comb line belonging to the bunch *j* can be written as

2 $$\xi_{i,j} = (f_{{\text{p}}} - f_{i,j} ) - \left\lfloor {\frac{{f_{{\text{p}}} - f_{i,j} }}{\delta }} \right\rfloor \delta ,$$

where *f*_*i,j*_ is frequency of a comb line and the bracket represents floor operation. Owning to the natively spaced nature, comb lines in the same bunch share identical offsets, so that offset of bunch *n* can be expressed as

3 $$\xi_{n} = n\Delta - \left\lfloor {\frac{n\Delta }{\delta }} \right\rfloor \delta .$$

Therefore, frequency distance between the merging comb lines in one resonance can be calculated from the offset difference of adjacent bunches

4 $$\Delta \xi = |\xi_{n} - \xi_{n - 1} | = \Delta - \left\lfloor {\frac{\Delta }{\delta }} \right\rfloor \delta .$$

This behavior is visualized in Fig. 1g, where offsets of three bunches (in the dashed box of Fig. 1f) are plotted relative to their mode numbers. When sending the sub-comb into a PD, inter- and intra-resonance beating would result in frequency components residing at higher and lower frequency, respectively (upper panel of Fig. 1h). At lower frequency region, beat-note Δ*ξ* and its harmonics can be observed, while at higher frequency region, beat-notes are symmetrically situated around *δ*, with the same spacing Δ*ξ*.

When consider adsorption of polar gases, which accept electrons (such as or H_2_S, NO_2_ and SO_2_) or offer electrons (such as NH_3_) to form π-π bonds with graphene. This variation of electron number of graphene will lead to change of its carrier density, modify graphene’s Fermi level [28], and interfere its conductivity [29]. This would further lead to change of permittivity and effective refractive index, then sequentially modifies GVD of graphene, and finally results in the alteration of Δ [30]. Although both *n*_g_ and *β*_2_ have impact on Δ*,* the contribution of *β*_2_ is dominant, since it is in the denominator, as shown in Eq. (1). In addition, slight change of *β*_2_ would bring about significant variation of Δ, which has been experimentally demonstrated in previous work [30]. Due to the aforementioned relation between Δ and beat-note Δ*ξ* in Eq. (4), gas adsorption can be eventually reflected by the frequency shift Δ*f* of the RF signal (or the sub-comb self-beating note), as shown in lower panel of Fig. 1h. As for non-polar gases, such as CO_2_, the interplay process is very similar. The only difference is that the bond they form with graphene is less stable, therefore response for them would be slightly less sensitive [18].

The comb dynamics and field evolution can be described succinctly by using the Lugiato-Lefever equation (LLE) [31],

5 $$t_{{\text{R}}} \frac{\partial E(t,\tau )}{{\partial t}} = [ - \frac{\kappa }{2} - {\text{i}}\delta \omega_{0} - {\text{i}}L\frac{{\beta_{2} }}{2}(\frac{\partial }{\partial \tau })^{2} + {\text{i}}\gamma L|E(t,\tau )|^{2} ]E(t,\tau ) + \sqrt \theta E_{{{\text{in}}}} .$$

Here, *t*_R_ represents the roundtrip time, and *E*(*t*, *τ*) is the intracavity electrical field with *t* and *τ* representing slow and fast time, respectively. On the right side of equation, *κ* is the total intracavity power loss, *δω*_0_ is phase detuning, expressed as *δω*_0_ = *ω*_0_ – *ω*_p_, *ω*_0_ and *ω*_p_ are angular frequencies of the pumping resonance and the pumping laser, respectively. *L* is the cavity length, *β*_2_ is the GVD, here higher-order dispersions are neglected. *γ* is the nonlinear coefficient, *θ* represents the coupling power loss, *θ* = 0.5*κ* when critical coupling is considered, and *E*_in_ is the electrical field of the launched-in pump.

**Fig. 2:**
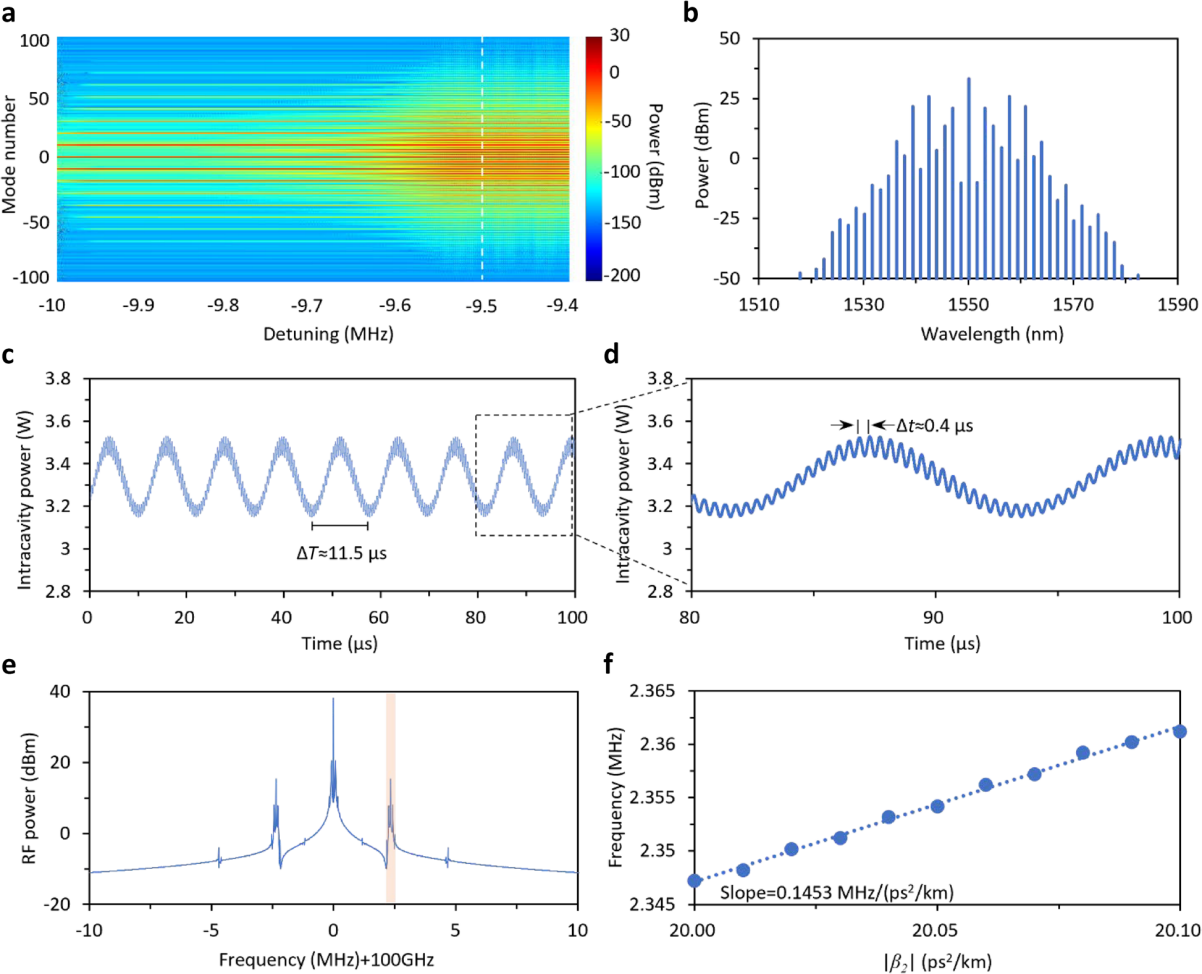
Numerical simulation. **a** Spectral evolution, here we scan the pump detuning from − 10 to − 9.4 MHz. Color bar: intracavity comb power. **b** Simulated sub-comb spectrum when the detuning is − 9.5 MHz, this spectrum is indicated by using the white dashed line in **a. c** Intracavity power trace over 100 μs when the detuning is fixed at − 9.5 MHz, showing a period of 11.5 μs. **d** Zoom-in of the intracavity power trace in **c**, it reveals structure with a smaller period of 0.4 μs. **e** Radio frequency spectrum at the repetition frequency, calculated from the intracavity power trace recorded over 10^7^ roundtrips, based on FFT. The sub-comb merging produces a signal with 2.5 MHz offset and small sidebands with frequency offset about 0.8 MHz, corresponding to the fast and slow oscillating periods, respectively. The peak in the shaded region is exploited as probe for sensing. **f** Correlation between the probe’s frequency offset and the GVD intracavity. To enhance the frequency resolution, intracavity power over 10^8^ roundtrips are recorded, enabling a resolution down to 1 kHz. Linear fitting is conducted, revealing slope equal to 0.1453 MHz/(ps^2^/km)

To delve the sub-comb dynamics and corroborate the sensing principle, we set the parameters based on our microsphere aforementioned. The FSR is set to be 100 GHz, *Q* = 5 × 10^8^, *β*_2_ =  − 20 ps^2^/km, *γ* = 1.79 × 10^−2^ W^−1^m^−1^ [32], and the launched-in pump power is set to be 20 mW. To have a better understanding of sub-comb formation process, we simulate the spectral evolution with respect to frequency detuning from − 10 to − 9.4 MHz (Fig. 2a). Since the long lifetime of photon in the high *Q* cavity leads to a slow field evolution, a Turing field is injected into the cavity at the beginning, to initiate the process and reduce the roundtrips for field build-up. It is evident that with detuning reduced (absolute value), secondary sidebands appear around primary lines, meanwhile comb bandwidth is broadened. The optical spectrum of comb state at detuning of − 9.5 MHz is demonstrated in Fig. 2b, and manifests itself as a sub-comb. After that, detuning is fixed at − 9.5 MHz, and intracavity power trace is recorded over 100 μs (1 × 10^7^ roundtrips) after intracavity field reaching stationary, as shown in Fig. 2c. The temporary trace shows a period Δ*T* ≈ 11.5 μs. After zoom-in, it displays a local period Δ*t* ≈ 0.4 μs (Fig. 2d). Through Fourier transform, corresponding RF spectrum can be obtained (Fig. 2e). The beating signal and its harmonics, rising from merging, spaced with an interval of 2.35 MHz. Besides, smaller sidebands reside around these peaks with 0.8 MHz spacing are also observed, whose generation can be attributed to the incoherence nature of sub-comb. These frequency components correspond to different periods in temporary domain. To unveil the impact of the change in *β*_2_ (which is related to *n*_g_), the peak at 2.35 MHz is selected as the probe. Another 10^8^ roundtrips are applied to calculate the RF spectrum, which ensures frequency resolution down to 1 kHz. By tuning GVD from − 20 to − 20.1 ps^2^/km, position of the probe shifts from 2.347 to 2.361 MHz. Their correlation is shown in Fig. 2f. Through linear fitting, the slope of the fitted trace is found to be 0.1453 MHz/(ps^2^/km), revealing that the merging signal could be sensitive to external gas adsorption.

## Experimental results

**Fig. 3:**
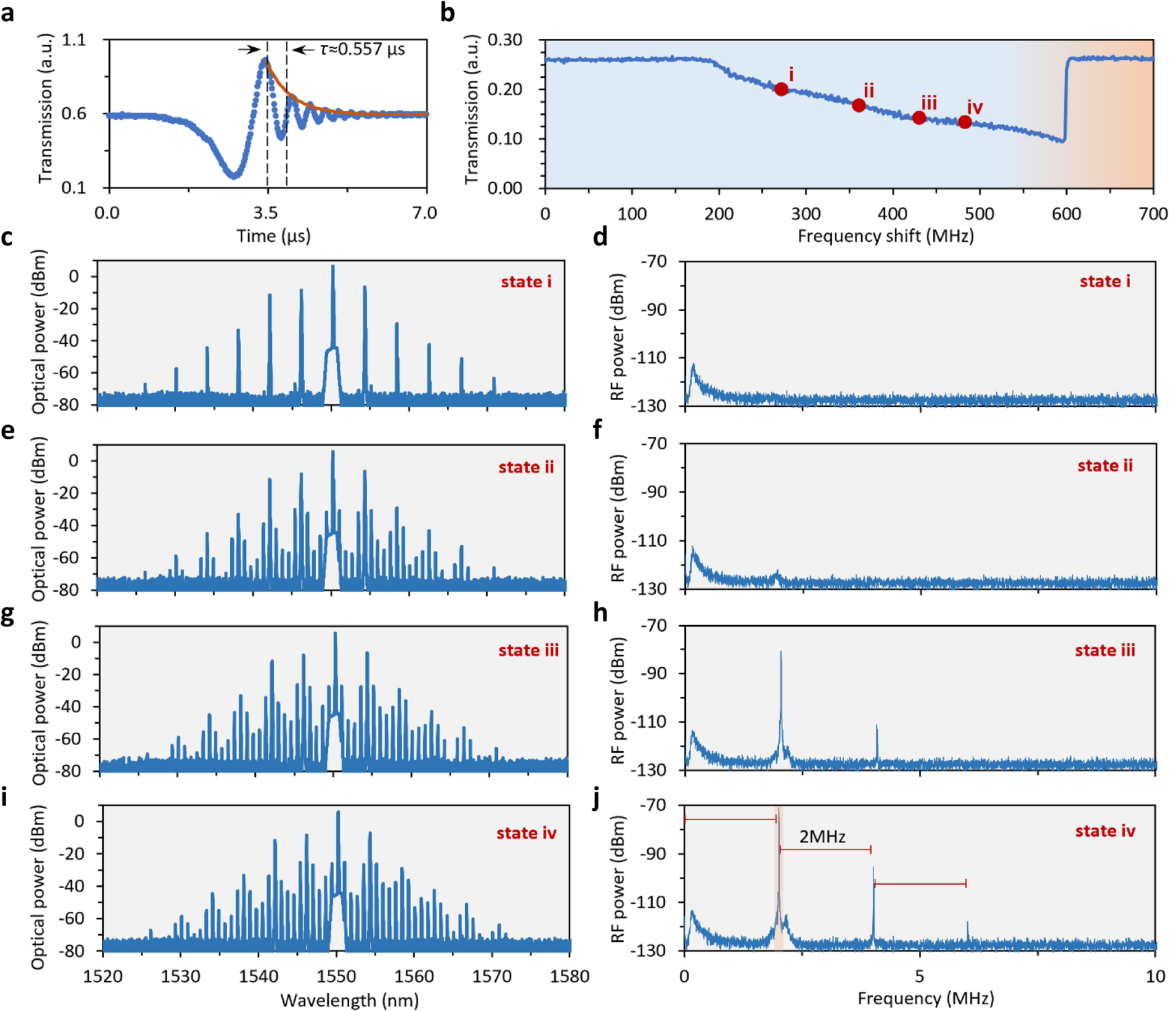
Generating sub-combs and measuring their beating signals. **a** Measured transmission of the pumping resonance at 1550 nm, which shows a ring-down profile under a sweeping speed of 10 nm/s. Exponential fitting reveals the photon lifetime is 0.557 μs, corresponding a loaded *Q* factor of 6.77 × 10^8^. **b** Measured comb evolution trace, when scanning the pump frequency in a range of 0 to 600 MHz. This scanning range covers the whole resonance. Here states i − iv are marked. **c − j** Optical spectra and radio frequency spectra of states i − iv. Resolution bandwidth (RBW) of the radio measurement is 100 Hz. In state iii and state iv, signals with 2 MHz spacing are generated, due to the sub-comb merging effect

First, we verify the *Q* factor of our graphene sensitized microresonator. Figure 3a shows the transmission of a resonance at 1550 nm. In this measurement, a tunable laser (Santec TSL-710) sweeps with a speed of 10 nm/s and a laser power of 0 dBm, which is below the nonlinear threshold. Under this condition, the laser frequency tuning within the resonance is commensurate with the photon lifetime, leaving a ring-down profile [33]. By finding the maxima of the oscillation amplitude, an exponential fitting is conducted. The photon lifetime can be approximated as the time needed to decay to e^−1^ level with respect to the maximum of the fitting [34], estimated to be 0.557 μs in our case. In this way, the loaded *Q* factor can be calculated in the equation *Q*_L_ = *ω* × *τ*, where *ω* is the angular frequency of resonance, and *τ* is the photon lifetime. As a result, loaded *Q* of this resonance is 6.77 × 10^8^, revealing the high *Q* preponderance of microsphere. In sub-comb generation and sensing, we use this resonance as the pumping resonance. After that, a narrow linewidth tunable laser (NKT-E15, typical linewidth 100 Hz) is used for comb generation.

With a launched-in power of 20 mW, we sweep the pump’s frequency across the resonance. Transmitting power of the pump during the scan is shown in Fig. 3b. Due to thermal effect, the resonance profile manifests itself as a typical thermal triangle shape. Here we mark four states in the evolution process, and plot their correspondent optical and self-beating spectra in Fig. 3c − j. In state i, primary sidebands are generated in optical spectrum, and we observe a flat radio frequency spectrum. The hump situated around 0.2 MHz is induced by PD (Thorlabs B570C). By further decreasing the pump-to-resonance detuning (increasing the pumping wavelength), secondary sidebands emerge and sub-combs appear (state ii). But in this state, the power of sub-combs is low, so we cannot see an obvious beating peak. With further red-detuning the pump, strong beat-note signals are generated, which is the key characteristic of the sub-comb merging (state iii). Finally, when the pumping frequency scan reaches 480 MHz, it comes to state iv, here a higher intracavity pump power leads to the generation of more harmonics, with equal spacing 2 MHz. This number critically refers the comb lines’ distance in one resonance. These beating signals demonstrate a maximum SNR up to 50 dB. In the sensing application next, we use the beating line with frequency 2 MHz (shaded region, with the highest SNR) as the sensing probe.

**Fig. 4:**
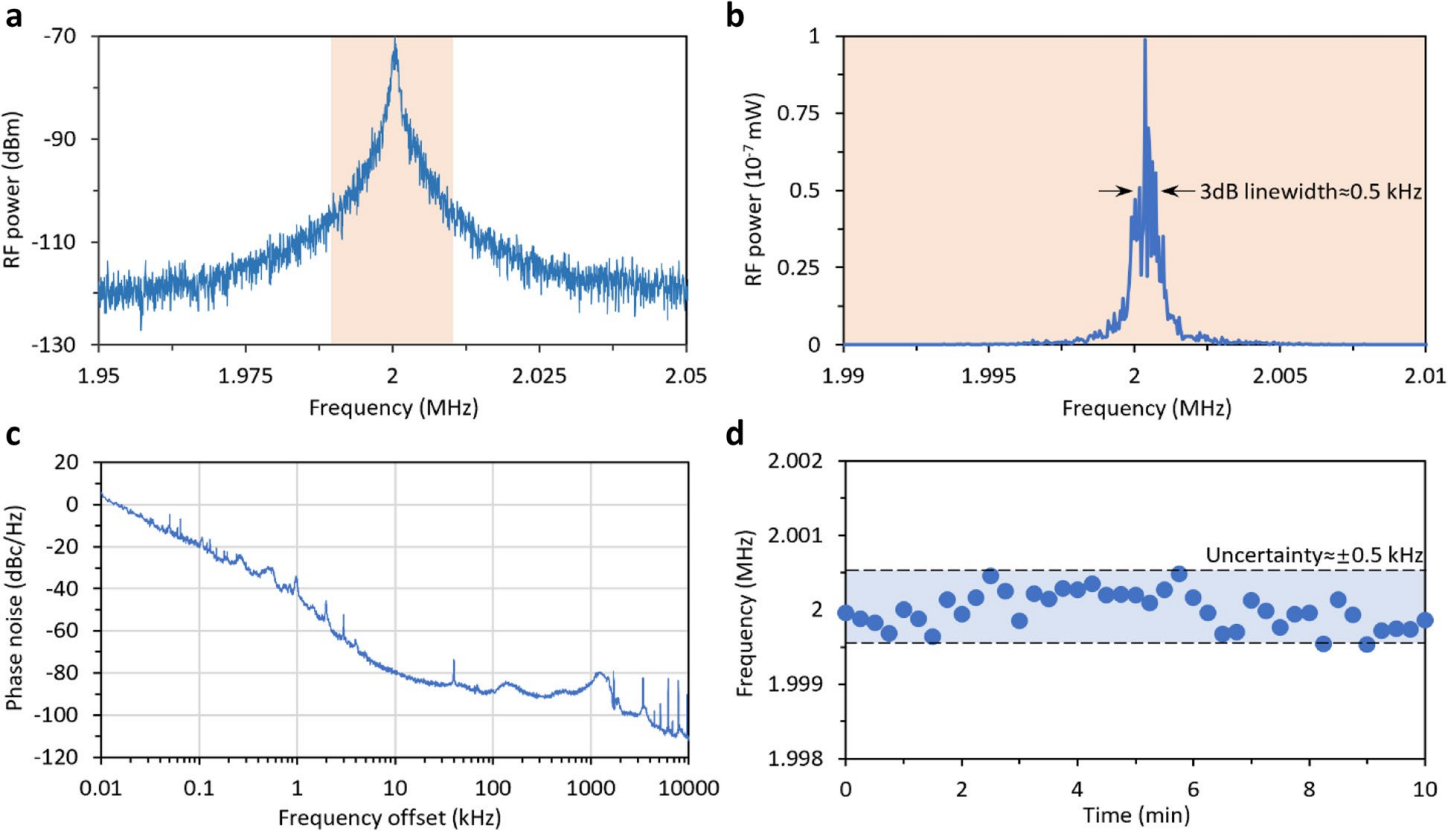
Probe signal characterization. **a** RF spectrum of the probe signal at 2 MHz. **b** Zoom-in of the probe. By plotting RF power in linear scale, its 3dB linewidth is evaluated, about 0.5 kHz. RBW of the spectrum is 10 Hz. **c** Single-sideband phase noise of the probe. Bandwidth (BW) = 0.1%. **d** Long-term frequency uncertainty of the probe in 10 min, showing an uncertainty of ± 0.5 kHz

In Fig. 4, we characterize the sensing probe by evaluating its linewidth and stability. The linewidth of a signal is crucial as it dictates the instantaneous spectral resolution, and the long-term drift affects the signal’s accuracy over time. A narrow linewidth coupled with high stability is typically sought after in sensing applications [11]. In our design, through sub-comb merging, the comb remains in a non-chaotic state, thus retaining the low noise features of the Turing state while offering a detectable low-frequency beacon, advantageous for demodulation in sensing tasks. Figure 4a provides a closer look at the sensing probe, with a display range of 0.1 MHz. At this scale, the noise floor is at − 120 dBm, and the peak power reaches − 70 dBm, confirming a signal-to-noise ratio (SNR) of 50 dB. In Fig. 4b, we further examine a 20 kHz segment (from 1.99 to 2.01 MHz) and present the signal in a linear scale. By directly reading its full width at half maximum, we establish that the 3 dB linewidth of this probe signal is approximately 0.5 kHz. This figure is significantly larger than the line-to-line beat note of a fully stabilized soliton [35], attributed to the sub-comb operating in a free-running mode. By applying an optoelectronic feedback technique [24], we anticipate that the linewidth could be further narrowed for various applications.

Figure 4c details the measured single sideband phase noise (SSB-PN) for the 2 MHz probe. Starting from 3 dBc/Hz at a 10 Hz offset, the SSB-PN values at various offsets are also provided, in details, they are − 19 dBc/Hz at 0.1 kHz offset, − 37 dBc/Hz at 1 kHz offset, − 80 dBc/Hz at 10 kHz offset, and − 87 dBc/Hz at 100 kHz offset. Finally, the curve demonstrates a − 82 dBc/Hz SSB-PN at 1 MHz offset. The SSB-PN trend follows a 1/*f*^*2*^ pattern in approximation, indicating that the primary source of instability is white noise. Moreover, we assess its long-term stability by tracking the peak position over an extended period, as illustrated in Fig. 4d. With a sampling time of 15 s, the peak frequency of the probe signal shows remarkable spectral stability across a 10-min duration, adequate for sensing data collection, with a measured uncertainty of about ± 0.5 kHz, mirroring its linewidth. These measurements affirm that the heterodyne probe, derived from the sub-comb merging effect, exhibits precise and stable performance, underscoring its potential for gas sensing applications in subsequent sections.

**Fig. 5:**
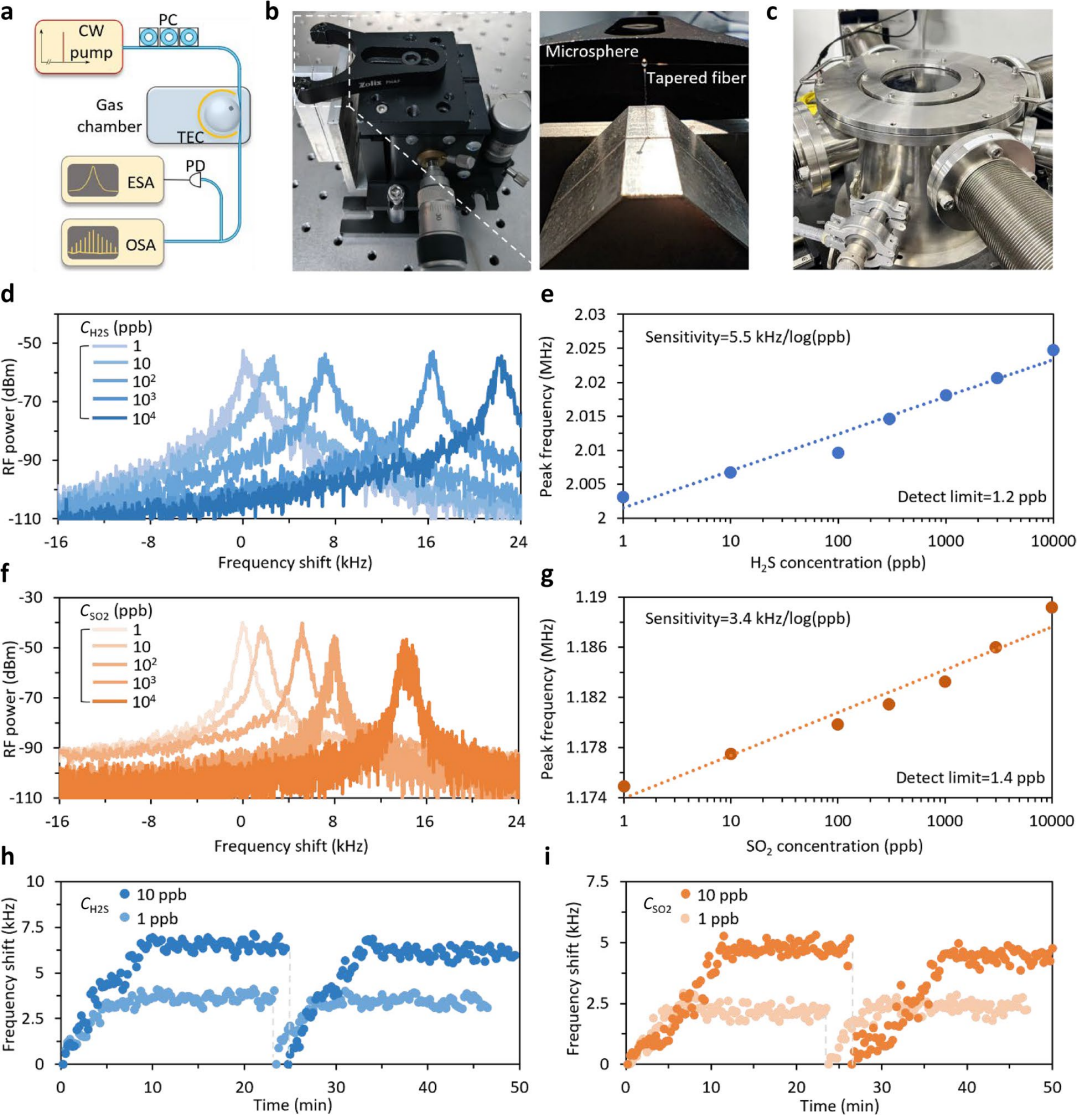
Gas sensing. **a** Experimental setup. **b** Picture of coupling system. **c** Picture of gas chamber. **d** RF spectra of probe signal under different H_2_S concentrations. The frequency shift is relative to its position at 1 ppb. **e** Correlation between H_2_S concentration and frequency of probe signal. **f** RF spectra of probe signal under different SO_2_ concentrations. **g** Correlation between SO_2_ concentration and frequency of probe signal. Detect limit is determined by frequency uncertainty (± 0.5 kHz) and sensitivity estimated from the slope of the fitted trace. **h** Response curve and stability of H_2_S sensing, measured under 1 and 10 ppb concentration. **i** Response curve and stability of SO_2_ sensing, measured under 1 and 10 ppb concentration

Finally, we apply the sub-comb merging effect to realize a high performance microcavity gas sensor, as Fig. 5 discusses. The experimental setup is illustrated in Fig. 5a. The graphene-sensitized microsphere macroscopically stabilized by a thermoelectric cooler (TEC), which can control local temperature with a resolution of 10 mK. Then the device is put in a gas chamber with a volume of 8 L. We use tapered fiber to couple light in and collect light out from the microresonator. In the sensing scenario, we use an external laser diode as pump, outputting 1550 nm light with optical power 20 mW. The polarization controller (PC) is used to optimize the SNR. Optical and electrical signals are measured in an optical spectral analyzer (OSA, Yokogawa AQ6370D) and an electrical spectral analyzer (ESA, Rohde & Schwarz FSW43) respectively. In this setup, we do not need an erbium-doped fiber amplifier, as *Q* factor of our graphene-silica microresonator has been already high enough, meanwhile sub-combs impose less requirement on pump power, in contrast to soliton state. Figure 5b shows pictures of the coupling system (left panel). The microsphere is fixed by using a customized clamp, while the position of tapered fiber is meticulously adjusted to just attach the equator (right panel), by leveraging a 3-axis compact flexure stage (Thorlabs, MBT616D/M). Then, the whole coupling system is deposited in a gas chamber (Fig. 5c).

Figure 5d shows the frequency shift of the probe signal, when injecting H_2_S gas with varied concentrations. Specifically, in air, frequency of the probe signal is 2 MHz (or, *f*_probe_ = 2 MHz). With increasing the concentration of H_2_S gas from 1 ppb to 10 ppm, *f*_probe_ blue shifts from 2.003 to 2.024 MHz. Specifically, when the concentration of H_2_S gas (*C*_H2S_) reaches 10, 100, 1k and 10k ppb, corresponding frequency shifts of the probe relative to peak position at 1 ppb are 3.61, 6.45, 14.94 and 21.64 kHz, respectively. The correlation between the probe frequency shift (Δ*f*_probe_) and H_2_S’s concentration is shown in Fig. 5e. By linear fitting, a sensitivity of 5.5 kHz/log(ppb) for H_2_S detection is suggested, which is estimated from slope of the fitted trace. Referring to both the frequency uncertainty of the *f*_probe_ (± 500 Hz) and sensitivity, this result demonstrates a detect limit down to 1.2 ppb for H_2_S detection, calculated as ratio of the two.

By exciting sub-combs in another transverse mode around 1550 nm, we can use this device to measure the concentration of another gas sample (e.g., SO_2_) simultaneously. We show the spectral shift of another sub-comb’s beat note in Fig. 5f. In this operation, the sub-comb merging generates a probe signal with initially residing at around 1.173 MHz. By increasing the concentration of SO_2_ gas (*C*_SO2_) from 1 ppb to 10 ppm, we record that the *f*_probe_ shifts from 1.175 to 1.189 MHz. In this case, when *C*_SO2_ values are 10, 100, 1k, and 10k ppb, spectral shifts of the probe relative to peak position at 1 ppb are 2.59, 4.92, 8.36, and 14.28 kHz, respectively. The evident degradation of the signal is due to detuning variation induced by frequency shift of the pumping resonance. The correlation between the probe frequency shift (Δ*f*_probe_) and SO_2_’s concentration is shown in Fig. 5g. It suggests a sensitivity of 3.4 kHz/log(ppb) for SO_2_ detection and also offers a measurable range over 10 ppm. Referring the frequency uncertainty of the *f*_probe_ (± 500 Hz) and sensitivity, this result demonstrates a detect limit down to 1.4 ppb for SO_2_ detection.

The discrepancy in the detection limits derived from two sensing targets can be attributed to two main factors. First, the quantity of electrons transferred between graphene and a gas molecule differs across the distinct gas samples. Schematically, to grasp a H_2_S molecule, graphene contributes two electrons, while to grasp a SO_2_ molecule, graphene just offers one electron. Consequently, the change in graphene’s GVD caused by the adsorption of a particular gas molecule is contingent upon the type of gas. Secondly, various transverse modes exhibit different degrees of overlap with graphene, leading to varying responses in signals produced by the same optoelectronic alteration in graphene. Although sensing for only H_2_S and SO_2_ is demonstrated, due to their wide existence and detrimental nature for both human health and apparatus operation, this sensor can actually be applied to any kind of gas, with also good sensing performance, according to the analysis of interplay mechanism.

Apart from sensitivity and detect limit, response time and reliability are also important indicators for a gas sensor. The response curves under both 1 and 10 ppb for each gas are recorded, and the measuring process is repeated for corroborating its stability. As Fig. 5h shows, when 10 ppb H_2_S injected into the chamber, frequency of the probe continuously shifts with gas diffusing in the air. After diffusion process completed, which consumes about 10 min, the frequency shift tends to be stabilized. Then, another 15 min are applied to record probe’s position, and the mean value during this section indicates that the final shift amount is 6.53 kHz. Subsequently, the gas is evacuated and air is refilled into the chamber, and the same procedure is conducted again. The averaged shift is calculated to be 6.12 kHz. The recoverability is 93.33%, estimated by final shifts of these two successive attempts. It does not completely recover, this can be explained as the incomplete desorption of gas molecules. As for 1 ppb H_2_S, after 5 min’ diffusion, the final shifts are 3.66 and 3.44 kHz respectively, with 93.60% recoverability, which is consistent with that of 10 ppb. Moreover, to validate the uniform performance of the sensor for different types of gases, the same experiment is conducted for SO_2_ (Fig. 5i). Under 10 ppb concentration of SO_2_, it takes about 11 min for the response curve to be flat, and the final shifts of two successive attempts are 4.75 and 4.43 kHz, which recover 93.27%. For 1 ppb SO_2_, the time for complete diffusion is 6 min, and the final shifts become 2.14 and 2.35 kHz, with 91.07% recoverability. The results illustrated above illustrate that the sensor owns reliable and steady response, regardless of gas type and concentration.

## Discussion and conclusion

In this study, we investigate the dynamics of sub-comb formation in a graphene-sensitized silica microsphere resonator and introduce a gas sensing scheme by exploiting the sub-comb merging effect. The close relationship between the sub-comb’s beating signal, endowed by merging, and the intracavity group velocity dispersion tunability is supported by both theoretical analysis and numerical simulation. Our experimental results confirm that such incoherent states, occurring during microcomb formation, produce remarkably stable beating signals characterized by both low phase noise and minimal long-term frequency uncertainty. Although the operation of the sub-comb outputs is straightforward, it was often overlooked in previous techniques. With graphene sensitization, this sub-comb heterodyne sensing device exhibits an exceptional response to gas molecular adsorption, achieving detect limits of 1.2 ppb for H_2_S gas and 1.4 ppb for SO_2_ gas, respectively. In summary, our research synergizes flexible comb formation, direct offset heterodyne detection, and graphene optoelectronics, leading to an easily operated and ultrasensitive miniature gas sensor. This exploration not only offers a simple system configuration but also sets a new standard for convenient optoelectronic detection. Looking ahead, beyond its application in microsphere-based gas sensing, our interdisciplinary approach shows promise for providing platform-independent solutions for a broader range of sensing applications, including on-chip biochemical sensing and photonic-microwave signal generation and control.

## Data Availability

The data that support the plots and maps within this paper and other findings of this study are available from the corresponding authors upon reasonable request.
